# Loop-mediated isothermal amplification assay as a sensitive diagnostic tool for *Leishmania donovani* infections in Sri Lanka

**DOI:** 10.4038/cmj.v61i2.8286

**Published:** 2016-06

**Authors:** H S Kothalawala, N D Karunaweera

**Affiliations:** 1 Department of Parasitology, Faculty of Medicine, University of Colombo, Sri Lanka.

**Keywords:** LAMP, cutaneous leishmaniasis, *Leishmania*, PCR, molecular

## Abstract

**Introduction:**

Cutaneous leishmaniasis (CL) in Sri Lanka is caused by *Leishmania donovani* MON 37. Confirmation of diagnosis is done through microscopy, either directly or after in vitro culture. Molecular diagnostic methods are sensitive, but require well established laboratories. Loop mediated isothermal amplification assay (LAMP) is rapid, specific for parasite species-specific DNA amplification, and requires only basic laboratory equipment. The aim of the study was to determine the potential utility of LAMP to diagnose leishmaniasis.

**Methods:**

Thirty one patients clinically diagnosed as CL were enrolled in the study. Light microscopy, a widely used and universally accepted method was used as the reference standard for confirmation of diagnosis.

**Results:**

LAMP was positive for 19/23 microscopically positive patients, yielding a sensitivity of 82.6%. Specificity of the LAMP assay was 100% and the positive and negative predictive values were 100% and 66% respectively. The average time taken for the LAMP assay was 1 hour and 40 minutes and the cost per sample was about SLR 2 000, which was approximately half the time and cost of a nested PCR (polymerase chain reaction).

**Conclusions:**

LAMP could be considered a potentially useful diagnostic tool for leishmaniasis.

## Introduction

Cutaneous leishmaniasis (CL) is considered as a neglected tropical disease [1]. A genetically distinct variant of *Leishmania donovani* is the causative agent in Sri Lanka (*L. donovani* MON 37) [2]. *Leishmania* spp. promastigotes are transmitted through female sandflies (*Phlebotomus argentipes*) during their blood meal. CL is the most prevalent clinical phenotype recorded in Sri Lanka, although a few cases of muco-cutaneous and visceral leishmaniasis have also been reported [3,4]. According to the database maintained at the Department of Parasitology, Faculty of Medicine, University of Colombo, about 3000 cases have been reported during the past decade. The definitive diagnosis of CL is important to provide specific treatment and plan effective disease control methods.

Diagnosis of cutaneous leishmaniasis is traditionally done by microscopy, which is the only established method in the endemic regions, though it requires services of skilled technicians and microscopists for preparation and examination of the slides [5]. Furthermore, microscopy cannot be used for species identification and the level of accuracy depends on the parasite count and the competency of the microscopist. It remains regular practice in most diagnostic laboratories to combine microscopy with *in vitro* cultures, which increases the sensitivity [6]. However, due to the laborious nature of these techniques and the requirement of skilled technical personnel for performance, molecular methods have come to the forefront in the recent times. Polymerase chain reaction (PCR) is considered a sensitive and specific method for diagnosis of leishmaniasis. But, routine PCR is not a practical tool in resource poor settings due to high cost, requirement of well-established laboratory facilities and expensive reagents. In this backdrop, more cost-effective and simple diagnostic tools need to be developed for effective management and control of leishmaniasis in Sri Lanka.

Loop-mediated isothermal amplification (LAMP) is a recently established tool for detection of many diseases including leishmaniasis [7]. The LAMP assay is superior because of its relative simplicity in performance, low cost, easy interpretation, high level of reproducibility, and relatively rapid performance time. The objective of the study was to determine the usefulness of the loop-mediated isothermal amplification assay as a diagnostic tool for Leishmania donovani infections in Sri Lanka.

## Methods

A total of 31 patients with skin lesions that were suspected to be leishmaniasis who were referred to the leishmaniasis clinic at the Department of Parasitology, Faculty of Medicine from July 2014 to May 2015 for laboratory tests for confirmation of diagnosis, were enrolled in the study. The participants formed a random series. Slit-skin scrapings and/or lesion aspirates were collected from the lesions of the patients using a standard protocol. Giemsa-stained smears of lesion aspirates were examined under the microscope (x100 magnification). Microscopy, which is a universally accepted and widely used method for parasite detection, was taken as the reference standard for the comparison of results. The culture media were inoculated for *in vitro* growth of parasites as previously described [6]. Cultures were microscopically examined after the second day of incubation for the presence of parasites.

DNA was extracted from the lesion aspirates or from the parasite culture after two days of inoculation, with the use of QIAamp DNA blood mini kits (Qiagen, Hilden, Germany). The extracted DNA was eluted in a final volume of 60 μl AE buffer (Qiagen, Hilden Germany). Nested PCR was performed using previously described methods that detect *Leishmania* genus. DNA with R 221(5’GGTTCCTTTCCTGATTTACG3’) R332.

(5’GGCCGGTAAAGGCCGAATA3’) as primers for the first round; R223.

(5’TCCCATCGAACCTCGGTT3’) and R333 (5’AAAGCGGGCGCGGTGCTG3’) as primers for the nested PCR round [8]. Two microlitres of the extracted DNA and 3 μl of each R221 and R332 primers were used in a 25 μl PCR mix containing 0.2 mM dNTP, 3 μl of 10× buffers containing 2mM MgCl_2_, 5 mM KCl, Tris-HCl (pH 9), 2 mM and 1U of Taq polymerase. For the second nested reaction 1 μl of the first PCR product was used as a template in the presence of 3 μl of each R223 and R333. Reaction conditions were same for both the first and second nested PCR cycles. Thermal conditions were 96°C for 5 minutes and 25 amplification cycles of 96°C for 30 seconds, 52°C for 30 seconds, 72°C for 5 minutes and termination at 4°C.The product was visualised on a 2% agarose gel.

LAMP was performed using the original protocol with minor modifications [9]. A set of four primers specific for *L.donovani* kinetoplast minicircle DNA were used (GenBank accession no.Y11401) [9]. The LAMP reaction was performed using 25 μl of reaction mixture containing 40 pmol each of FIP and BIP primers, 5 pmol each of F3 and B3C primers,1.4 mM each of dNTP,0.8 M betaine, 10X bst amplification buffer (New England Biolabs, Ipswich, MA), containing 20 mM Tris-HCl, 10m M(NH4)_2_SO4, 10 mMKCl, 2 mM MgSO_4_, 0.1% Triton X-100, pH set for 8.8 at final 1× and additional 6 m M MgSO^4^ and 8 units of *bst* polymerase large fragment (New England Biolabs, Ipswich, MA). The reaction mixture was incubated at 62°Cfor 1 hour and 30 minutes and chilled on ice to visualize the precipitate. The tube was wrapped in paraffin prior to the LAMP assay to avoid contamination.

Sensitivity, specificity, positive predictive value (PPV) and negative predictive value (NPV) of each assay were calculated considering microscopy as the gold standard. Adjusted Walds method was used to calculate the confidence interval, which is considered as the most accurate method for small binomial samples [10]. Approval for the study was obtained from the Ethics Review Committee, Faculty of Medicine, University of Colombo.

## Results

Twenty three out of 31 patient samples were found to be positive for Leishmania parasites through microscopy (either direct or after culture). All the microscopy positive samples were also positive via nested PCR. The results of LAMP assay were interpreted depending on the presence or absence of turbidity through direct visualisation. Out of the 23 specimens that were positive through microscopy and PCR, 19 were positive for the LAMP assay.

The LAMP assay yielded a sensitivity of 82.6%. The specificity of the LAMP assay was 100% while the positive and negative predictive values were 100% and 66% respectively. Nested PCR on the other hand showed 100% sensitivity, specificity, PPV and NPV values. The 95% 2-tailed confidence interval of a success rate for the LAMP assay as calculated by this test was 0.70–0.95, while the values for nested PCR and the microscopy were 0.87–1.02. The overlap between these values indicated that results were not significantly different.

The average time taken for the LAMP assay was 1 hour and 40 minutes when compared to the nested PCR that took approximately 3 hours and 30 minutes. The cost analysis was done for the determination of the cost-effective method between LAMP and nested PCR. The cost of performing LAMP was SLR 2000 per sample and the cost of performing the nested PCR per sample was SLR 3560. The cost was calculated with all the reagents used for the methods including the shipping cost, but the cost calculation excluded the initial investment made for laboratory equipment, such as water bath, pipettes, eppendorf tubes, tips, PCR machine and the wages.

## Discussion

Cutaneous leishmaniasis is a newly established disease in Sri Lanka, and still lacks a relatively simple, low cost and a reasonably sensitive diagnostic tool that can be performed with minimal technical expertise.

LAMP is a technique used for the diagnosis of many infectious diseases, such as malaria and leishmaniasis [9,10]. The LAMP reaction is used for genetic testing, point of care testing, testing food products and environmental sample testing [12]. Performance indices of the LAMP assay tested under local conditions are indeed encouraging, but with clear room for improvement of sensitivity levels, though the results obtained are similar to the values reported in earlier studies [9, 13]. The specificity and the positive predictive value of the LAMP assay were similar to both the microscopy and nested PCR. This is probably due to the four primers used in this assay system that target six distinct regions within the parasite genome. It is also known that the use of *bst* polymerase that gets activated during higher temperatures minimises non-specific bands, and has the potential to resist inhibitors, which occur during nested PCR [11]. Unlike microscopy, the LAMP assay has the distinct advantage of identifying the causative organism up to species level, which could be of particular importance in the management of atypical clinical manifestations of leishmaniasis, as is widely evident in Sri Lanka, and for epidemiological studies [3].

The LAMP assay has other advantages such as relative simplicity in performance, low cost, easy interpretation, high level of reproducibility, and relatively rapid performance time. The nested PCR assay, on the other hand, is only genus-specific (similar to microscopy) and requires expensive equipment, highly trained personnel, stringent laboratory conditions and longer performance time. LAMP technique could be considered particularly attractive in the local setting since it requires only a heat block or a simple water bath, in contrast to the expensive PCR thermo cyclers, gel documentation systems and UV transilluminator that are required for regular molecular assays. Furthermore, the assay is conducted under isothermal conditions for a shorter time, which is known to minimise inhibitions that generally occur during the latter stages of the PCR cycles, that could interfere with performance.

The LAMP technique could be performed even in places without electricity, since the power supply to the water bath can be replaced with alternative methods such as batteries or solar systems. The result of the LAMP assay is interpreted through visualisation of turbidity, bypassing the need for post-PCR analysis involving gel electrophoresis, which is laborious and has the risk of product contamination, possible errors in interpretation, as well as potential exposure to toxic agents. Sensitivity and negative predictive value of LAMP assay, were less than with either microscopy or nested PCR. So, a negative result in the LAMP assay, particularly in a patient who is strongly suggestive of leishmaniasis based on clinical grounds, might require nested PCR as a second-line investigation to confirm or refute the diagnosis. The small sample size used in our study is a limitation.

In conclusion, the LAMP assay optimised for use under local conditions, appears to be a useful tool for diagnostic laboratories in Sri Lanka for the confirmation of leishmaniasis.
